# Supplementary material for: Principles of self-organization and load adaptation by the actin cytoskeleton during clathrin-mediated endocytosis
Source: eLife. 2020 Jan 17;9:e49840. doi: 10.7554/eLife.49840 (PMC7041948; doi:10.7554/eLife.49840)
Supplement: Supplementary file 1. [file elife-49840-supp1.docx]

| Membrane tension [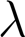](https://www.codecogs.com/eqnedit.php?latex=%5Clambda%250)(pN/nm) | Spontaneous curvature [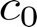](https://www.codecogs.com/eqnedit.php?latex=c_%7B0%7D%250) ([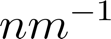](https://www.codecogs.com/eqnedit.php?latex=nm%5E%7B-1%7D%250)) | Length scale [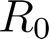](https://www.codecogs.com/eqnedit.php?latex=R_%7B0%7D%250)  (nm) | Non-dimensional membrane area  [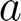](https://www.codecogs.com/eqnedit.php?latex=a%250) | Non-dimensional coat area [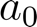](https://www.codecogs.com/eqnedit.php?latex=a_%7B0%7D%250) | Bending rigidity [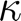](https://www.codecogs.com/eqnedit.php?latex=%5Ckappa%250) (pNnm) | Coat rigidity/ Membrane rigidity [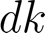](https://www.codecogs.com/eqnedit.php?latex=dk%250) | [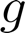](https://www.codecogs.com/eqnedit.php?latex=g%250) |
| --- | --- | --- | --- | --- | --- | --- | --- |
| 0.051 - 0.451 | 0.02 | 20 | 200 | 7 | 320 | 7.5 | 20 |
