# Supplementary material for: Principles of self-organization and load adaptation by the actin cytoskeleton during clathrin-mediated endocytosis
Source: eLife. 2020 Jan 17;9:e49840. doi: 10.7554/eLife.49840 (PMC7041948; doi:10.7554/eLife.49840)
Supplement: Supplementary file 2. [file elife-49840-supp2.docx]

| Non-dimensional coat area [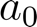](https://www.codecogs.com/eqnedit.php?latex=a_%7B0%7D%250) | [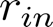](https://www.codecogs.com/eqnedit.php?latex=r_%7Bin%7D%250) (nm) | [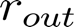](https://www.codecogs.com/eqnedit.php?latex=r_%7Bout%7D%250)  (nm) | [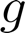](https://www.codecogs.com/eqnedit.php?latex=g%250) |
| --- | --- | --- | --- |
| 7 | 100 | 200 | 20 |
