# Supplementary material for: Principles of self-organization and load adaptation by the actin cytoskeleton during clathrin-mediated endocytosis
Source: eLife. 2020 Jan 17;9:e49840. doi: 10.7554/eLife.49840 (PMC7041948; doi:10.7554/eLife.49840)
Supplement: Supplementary file 3. [file elife-49840-supp3.docx]

| Parameter | Rate | Reference | Note |
| --- | --- | --- | --- |
| Arp2/3 binding rate | 7/s | Beltzner *et al.* 2008 | Assumes 100 nm long filaments |
| Unbinding rate | 0.001/s | Beltzner *et al.* 2008 |  |
| Nucleation rate | 1/s | Beltzner *et al.* 2008 |  |
| Branching angle | 77 ± 13˚ | Blanchoin *et al.* 2000 |  |
| Actin persistence length | 10 µm | McCullough *et al.* 2008 |  |
| Actin growth rate (+ end) | 182/s | Pollard 1986 | Assumes 20 µM ATP-actin |
| Actin stall force | 5 pN | Footer *et al.* 2007 | Assumes 20 µM ATP-actin |
| Capping rate | 3.5/s | Berro *et al.* 2010 | Assumes mean filament length is 150 nm |
| Hip1R affinity | 400nM | Engvist-Goldstein *et al.* (2001) |  |
| Hip1R number | 200 | Sirotkin *et al.* 2010 | Based on actin-pit linkers in yeast |
| Nucleator number | 30 |  |  |
| Arp2/3 number | 200 | This study |  |
| Membrane tension | 0.12pN/nm | Dai and Sheetz (1999)  Diz-Muñoz et al. (2016)  Kaplan *et al.* (in preparation) | Calibrated by membrane mechanics modeling |
